# The Synergistic Beneficial Effects of Ginkgo Flavonoid and *Coriolus versicolor* Polysaccharide for Memory Improvements in a Mouse Model of Dementia

**DOI:** 10.1155/2015/128394

**Published:** 2015-03-02

**Authors:** Xianying Fang, Yan Jiang, Hui Ji, Linguo Zhao, Wei Xiao, Zhenzhong Wang, Gang Ding

**Affiliations:** ^1^College of Chemical Engineering, Nanjing Forestry University, 159 Long Pan Road, Nanjing 210037, China; ^2^Jiangsu Key Laboratory of Biomass Based Green Fuels and Chemicals, 159 Long Pan Road, Nanjing 210037, China; ^3^Department of Pharmacology, China Pharmaceutical University, 24 Tongjiaxiang, Nanjing 210009, China; ^4^Jiangsu Kanion Pharmaceutical Co., Ltd., 58 Haichang South Road, Lianyungang 222001, China

## Abstract

This study reports the combination of Ginkgo flavonoid (GF) and *Coriolus versicolor* polysaccharide (CVP) in the prevention and treatment of a mouse model of Alzheimer's disease (AD). GF is a traditional health product, and CVP is the main active ingredient of the medicinal fungus *Coriolus versicolor*. The Morris water maze test, the Y maze, and the step-through test showed that the combinational use of CVP and GF synergistically improved memory in a mouse model of AD. Based on H&E staining analysis, the combination of CVP and GF decreased the severity of the pathological findings in the brain. Given that the expression of IL-1*β*, IL-6, and TNF-*α* was downregulated, the inflammation response in AD mice was considered to be inhibited. The downregulation of GFAP further demonstrated that inflammation was reduced in the brain of AD mice following treatment. Moreover, the expression levels of superoxide dismutase (SOD) and catalase (CAT) were elevated in the brains of treated mice, indicating that oxidation levels were reduced upon the combination treatment. Our results provide new insights into the efficient utilization of traditional medicine for preventing dementia.

## 1. Introduction 

Alzheimer's disease (AD) is the most common form of dementia, which worsens as it progresses and eventually leads to death. As a degenerative disease of the brain, AD is characterized by the sustained nervous disorders of the activities and functions of the brain [1]. For AD patients, there are obstacles of memory thinking, analysis judgment, visual identity, emotions, and so on. As the population ages, the prevalence of AD and related dementias is also increasing [2].

Neuroinflammation is involved in the onset of several neurodegenerative disorders [3]. Microglia cells are macrophage and representative of the innate immune system in brain. AD brain is marked by obvious inflammatory features, in which microglial activation is the driving force [4]. Astrocytes are the most abundant type of glial cells in the central nervous system (CNS) and are capable of accumulating substantial amounts of neuron-derived, amyloid *β*(1–42) (A*β*42)-positive material and other neuron-specific proteins [5]. Glial fibrillary acidic protein (GFAP) is expressed in astrocytes and is usually activated during the inflammation caused by AD [6–8]. In addition, cellular oxidation is also a main cause for aging [9]. Organisms have well-developed defense systems to eliminate reactive oxygen species (ROS). However, AD patients usually show defects with radical scavenging.

A variety of AD models have been developed for the study of anti-AD effects. D-galactose (D-gal) is a physiological nutrient ingredient in human metabolic process. Rodents injected with D-gal for 6–10 weeks show progressive deterioration of learning and memory capacity [10]. Based on the hypothesis of “aluminum poisoning,” a number of studies have used aluminum chloride (AlCl_3_) to produce an animal model of AD [11]. Therefore, D-gal and AlCl_3_ induced dementia were widely adopted in AD study [12]. Classic drugs, such as acetylcholinesterase inhibitors (AChEIs), fail to decline disease progression and display several side effects that reduce patient's adhesion to pharmacotherapy [13]. Currently, more attention has been focused on the development of anti-AD drugs from Chinese Material Medica (CMM) for their multicomponent features, including the ability to affect multiple targets and levels signaling pathways [14].

Based on a wide range of preclinical effects demonstrated for Ginkgo biloba, Ginkgo biloba extract EGb 761 can be conceptualized as a multitarget compound with activity on distinct pathophysiological pathways in AD and age-related cognitive decline [15]. A standardised extract of Ginkgo biloba (EGB) leaves is a well-defined product and contains approximately 24% flavonoids and 6% terpene lactones [16]. Ginkgo flavonoids (GFs) are the main constituents in EGB, consisting mainly of flavonols such as quercetin, kaempferol, and isorhamnetin [17]. These GFs have free radical scavenging effects and could inhibit lipid peroxidation [18]. Other studies demonstrated that GF exhibited neuroprotective effects via antioxidant activity in brain damaged mice caused by ischemia-reperfusion [19].

Although multiple studies have suggested the cognition enhancing properties of Ginkgo flavonoid, the benefits of Ginkgo biloba are controversial [20, 21]. Therefore, rational formulations that include other natural active substances should be considered [22]. An extremely broad range of physiological effects has been linked with the use of* Coriolus versicolor* polysaccharide (CVP), including calming of the CNS [23, 24]. A recent study shows that CVP can reduce the lipid peroxidation level in brain tissues during exhaustive exercise in rats and can accelerate the removal of free radicals [25]. Considering the complementary pharmacological effects of CVP and GF on cognition system and the immune system, we here study the complex effects of a combination treatment with these two compounds in a mouse model of AD.

## 2. Materials and Methods

### 2.1. Reagents

Ginkgo flavonoid and* Coriolus versicolor* polysaccharide were purchased from Xian Reain Biotechnology Co., Ltd. (Xian, China). D-gal was purchased from Shanghai Huixing Biochemical Reagent Co., Ltd. (Shanghai, China). Galantamine hydrobromide tablets (8 mg/tablet) were purchased from Xian Janssen Pharmaceutical Ltd. (Xian, China). Anhydrous AlCl_3_ was purchased from Sinopharm Chemical Reagent Co., Ltd. (Shanghai, China). Morris water maze was purchased from Shanghai Jiliang Software Technology Co. Ltd. (Shanghai, China). Y maze and step-through test instrument was purchased from Huaibei Zhenghua Biologiques Equipment Co. Ltd. (Anhui, China). Real Envision Detection kit was purchased from GeneTech Company (Nanjing, China). Lysis buffer and PMSF were purchased from Beyotime (Nanjing, China). LumiGLO chemiluminescent system was purchased from KPL (Guildford, UK).

### 2.2. Animals

Six-week-old ICR mice (20 ± 2 g, male) were purchased from Comparative Medical Center of Yangzhou University (Yangzhou, China). They were maintained with free access to pellet food and water in plastic cages at 21 ± 2°C and kept on a 12 h light/dark cycle. Animal welfare and experimental procedures were carried out in accordance with the internationally accepted Guide for Care and Use of Laboratory Animals (Ministry of Science and Technology of China, 2006) and the related ethical regulations of Nanjing Forestry University. All efforts were made to minimize the animals' suffering and to reduce the number of animals used.

### 2.3. Animal Model and Drug Administration Procedures

Mice were divided into 6 groups randomly. Dementia model mice were supplied with AlCl_3_ (0.5 mg/mL) solution everyday as drinking water and were administered with D-gal (10 mg/mL, 0.1 mL/10 g) physiological saline solution intraperitoneally in the daily morning for 54 days.

Drug dose is as follows: control group and model group, water; galantamine group, 6 mg/kg/d; GF group, 75 mg/kg/d; CVP group, 90 mg/kg/d; CVP & GF group, 37.5 mg/kg/d of GF and 45 mg/kg/d of CVP. Drugs were administered every day in the afternoon by intragastric administration.

### 2.4. Morris Water Maze Test (MWM)

The MWM was a relatively simple procedure typically consisting of six-day trials. Mice were applied for visible platform training on the first two days and for hidden platform training on the following three days. Probe trial was taken on the last day. Tests were carried out 2 h after drug delivery.

For visible platform training, mice were transferred from their housing facility to the behavior room and kept in an area where they cannot see the pool or spatial cues. Mice were allowed to adjust to the new environment for at least 30 min before testing. A flag (5 cm higher than the platform) was place in the fourth quadrant. Water of 25°C was added into the pool until the surface was kept 5 mm higher than the platform. Each mouse was brought to the testing area facing the edge of the pool and was allowed to swim for 90 s. If the mouse finds the platform before the 90 s cut-off, allow the mouse to stay on the platform for 10 s and then return it to its home cage. The escape latency was monitored and recorded by the computer. If the mouse does not find the platform, place the mouse on the platform and allow it to stay there for 30 s, and the escape latency was recorded as 90 s. The trial was repeated with four different starting quadrants.

For hidden platform training, a platform without flag was placed at the fourth quadrant. Water of 25°C was added into the pool and the surface was kept 5 mm higher than the platform. The training method was similar with the visible platform.

For probe trial, the platform was removed from the pool. Mice were brought to a selected quadrant as before and were allowed to swim freely for 90 s. The percent time and passing times of mice traveled into the forth quadrant were recorded. During the behavior test, the light was kept soft, environment was kept quiet, and references were kept in the same position.

### 2.5. Y Maze Test

The Y maze was constructed of black plastic walls 10 cm high. It consisted of three compartments (10 cm × 10 cm) connected with 4 cm × 5 cm passages. Y maze was a test of two-day trial, consisting of study training on the first day and testing on the second day, and was taken 2 h after intragastric administration. The mouse was placed in one of the compartments and allowed to move freely for 5 min. An arm entry was set as the starting point and mice were supplied with electric shock manually when all four paws entered the compartment. A one-time escape to the safe area was recorded as the right response. The test was repeated until the mice escaped to the safe area. The training was repeated 10 times with an interval of 30 s. After testing of each mouse, the maze was thoroughly cleaned to standardize odors. No adjustment stage was given on the second day. Ten tests were taken in each mouse and number of the right response was recorded.

### 2.6. Step-Through Test

The test cage is divided into the two compartments, the light and the dark by the guillotine door. The animal is placed in the light compartment and allowed to explore there for a preset period of time so that it may be familiar with its environment. Then the door is opened and it tries to get in the dark room since it prefers a dark place. As soon as it gets into the dark, an electrical shock is given manually. It was also a test of two-day trial, consisting of study training on the first day and testing on the second day, and was taken 2 h after intragastric administration. The time taken by the mice to meet the electric shock in the dark compartment since it was transferred to the light compartment was recorded as the latency. If the mouse does not step into the dark compartment, the latency can be identified as 300 s. Numbers of the mouse stepping into the dark compartment in 5 min were recorded as error numbers.

### 2.7. H&E Staining

Paraffin sections of the brain tissue were produced successively by fixation with 10% formaldehyde, dehydration, infiltration with paraffin, embedding, and slicing. Sections were infiltrated in distilled water for 5 min and the nuclei were stained with haematoxylin. After being rinsed in running tap water, sections were differentiated with 0.3% acid alcohol. Then, the sections were rinsed successively in running tap water, Scott's tap water substitute, and tap water. Finally, the sections were stained with eosin for 2 min, dehydrated, cleared, and mounted. Pictures were taken by the light microscope.

### 2.8. Immunohistostaining (IHC)

Paraffin-embedded brain sections were stained with 1 : 200 dilution of immunohistochemistry (IHC) primary antibody. Detection was done using Real Envision Detection kit. The images were collected with microscopy (BX51TRF; Olympus).

### 2.9. Western Blotting (WB)

WB analysis was taken as previously described [26]. In brief, the brain tissue was collected and made into homogenate with a 1 : 9 dilution with lysis buffer (PMSF added). The supernatant was collected by centrifugation for 10 min at 4°C, 12,000 r/min. Proteins were fractionated by SDS-PAGE and electrotransferred to PVDF membranes. Antibodies to GFAP and *β*-actin were used for blotting, and detection was done by LumiGLO chemiluminescent system.

### 2.10. Quantitative Analysis of Gene Transcription (Q-PCR)

Total RNAs were extracted using the procedures described previously [27]. Total RNA was reverse-transcribed into cDNA using the PrimeScript RT Reagent Kit (TaKaRa, Japan). For quantitative RT-PCR analysis, amplification was carried out for 40 cycles of the PCR conditions according to the instruction of SYBR Green I dye. Reactions were run in triplicate using *β*-actin as the internal RNA control on an ABI 7000 Thermocycler (Applied Biosystems Inc., Foster City, CA). The relative expression levels of the different genes were calculated using the 2^−ΔΔCT^ method.

### 2.11. Statistical Analysis

Data are expressed as mean ± SEM. Student's *t*-test and one-way ANOVA test were used for statistical analyses of the data. All statistical analyses were conducted using SPSS 10.0 statistical software (SPSS, Chicago, IL). Cases in which *P* values of <0.05 were considered statistically significant. *F* values with the degree of freedom (DF) were also provided. The exact probability levels were provided in the Supplementary Material available online at http://dx.doi.org/10.1155/2014/128394.

## 3. Results

### 3.1. The Combination of CVP and GF Shows Beneficial Synergistic Effects on the Learning and Memory Capabilities of AD Mice

**Figure 1:**
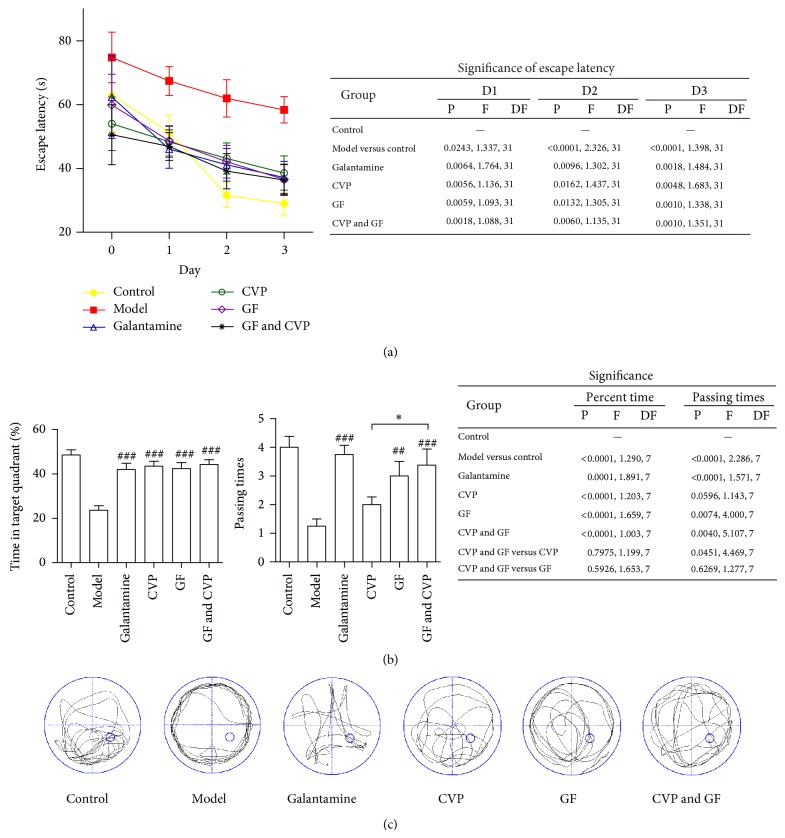
The CVP and GF treatment paradigm synergistically improves spatial learning and memory abilities in the MWM in a mouse model of dementia. The ICR mice (*n* = 8) were supplied with AlCl_3_ (0.5 mg/mL in water) and intraperitoneally injected with D-gal (10 mg/mL in physiological saline, 100 mg/kg/d). Fifty-four days later, the mice were tested in the MWM. (a) After two days of visible platform training, the mice were subjected to the hidden platform test, and the escape latency was recorded. Statistical analysis was performed to determine the *P* and *F* values* versus* the model group unless otherwise specified. (b) In the probe trial on the 6th day, the percent time that the mice spent in the fourth quadrant, which is where the hidden platform was previously placed was recorded. The number of times the mice passed through this quadrant was also recorded. Statistical analysis was performed to determine the *P* and *F* values* versus* the model group unless otherwise specified. ^*^ *P* < 0.05; ^##^ *P* < 0.01; ^###^ *P* < 0.005* versus* model group. (c) The swim-tracking paths employed by mice in the probe trial were recorded. The small blue circle indicates where the hidden platform was previously placed.

MWM was established to test hippocampal-dependent learning, including acquisition of spatial memory and long-term spatial memory. The MWM is a relatively simple procedure typically consisting of six-day trials, the main advantage being the differentiation between the spatial (hidden platform) and nonspatial (visible platform) conditions [28]. In the first two days (visible platform trials), there is no difference between the drug treated and control groups in latency (data not shown) indicating that both of the groups have similar visual and exercise abilities. We deemed that the mice are able to see the flagged-platform and the cues in surrounding environment and can swim acceptably. In the following 3 days (hidden platform trials), the escape latency of mice in model group was prolonged significantly according to the normal control group and was shortened significantly by the combination treatment of CVP and GF (Figure 1(a)). The probe trial results on the last day (Day 6) showed that the percent time and number of times the mice traveled into the forth quadrant, where the hidden platform was previously placed, were more in CVP & GF group than model group (Figure 1(b)). The swim-tracking paths revealed the searching strategies employed by mice (Figure 1(c)). The statistical significances of *P* value* versus* model group in CVP & GF group were all smaller than in single-drug groups. And the significant difference of passing time between CVP and CVP & GF group was significant in the probe trial. These data indicated that the combination treatment of CVP and GF synergistically improves the memory deficits observed in AD mice.

**Figure 2:**
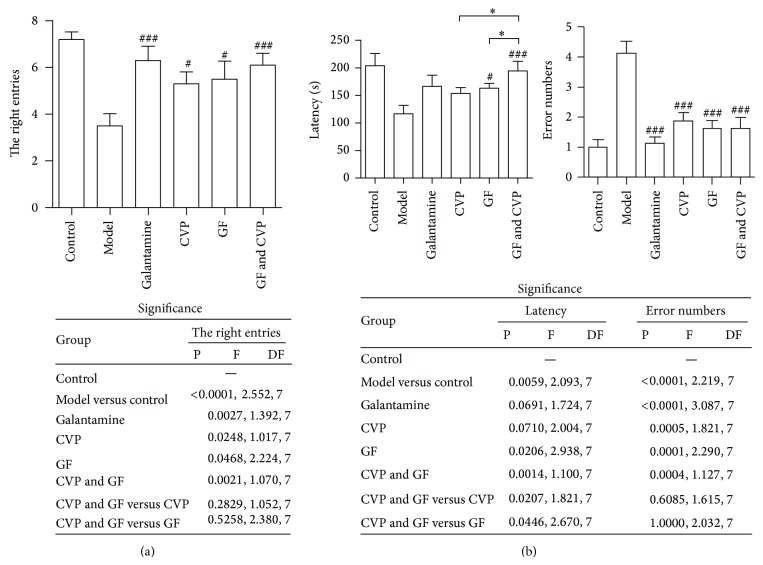
The Y maze and the step-through test were conducted to evaluate the synergistic effects of CVP and GF on learning and memory functions. (a) Following one day of training, the mice were tested in a Y maze 2 h after drug administration and the total number of right arm-entries during the ten tests was recorded. (b) Following one day of training, the mice were subjected to a step-through test 2 h after drug administration. The latency and number of error in 5 min were recorded. Data are mean ± SEM of three independent experiments (*n* = 8). ^*^ *P* < 0.05; ^#^ *P* < 0.05; ^###^ *P* < 0.005* versus* model group.

Except for MWM test, Y maze and step-through test are also widely used for measuring the learning and memory abilities of AD mice through applying various stimuli [29, 30]. On the first day of Y maze training, there was no significant difference in the right entries of mice between each group (data not shown). On the second day of Y maze test, the right entries were increased significantly upon drug administration, especially in CVP & GF group (Figure 2(a)). On the first day of step-through test, there was no significant difference in the latency of mice between each group (data not shown). Results of step-through test on the second day showed that the entry latency was prolonged significantly upon combination treatment of CVP & GF, while the error numbers were reduced significantly (Figure 2(b)). The therapeutic effects between the single treatment of CVP or GF and the combination treatment of CVP & GF were significant.

### 3.2. The Combination of CVP and GF Synergistically Decreases the Severity of Pathological Findings in the Brain Tissue of AD Mice

**Figure 3:**
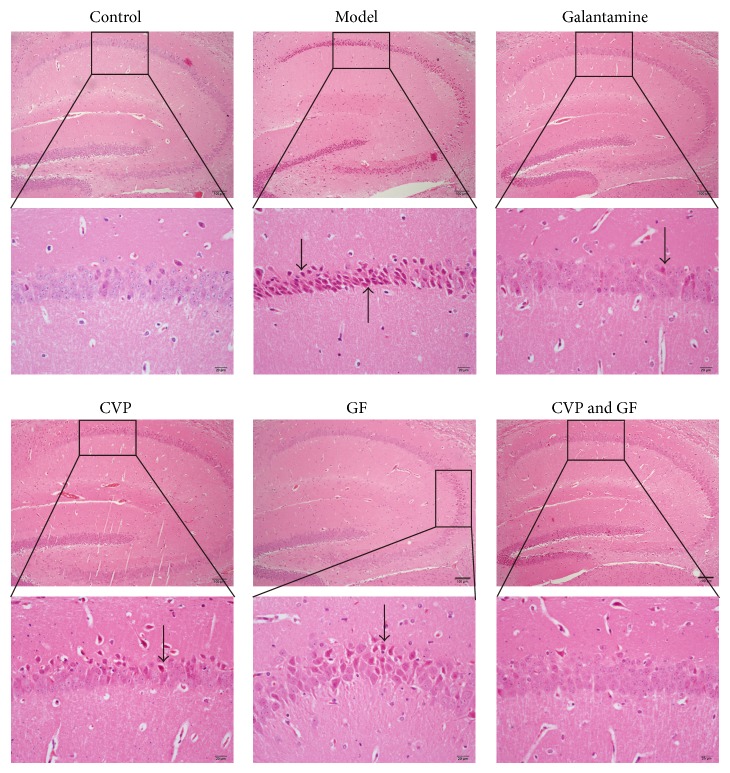
Histopathologic examination of the brain tissue. Paraffin sections of brain tissue from AD mice were analyzed by H&E staining (100x and magnified to 400x). Shrinkage, necrosis, and hyperchromatic nuclei were indicated by arrows. Data are representative of each group (*n* = 8). Scale bar, 100 *μ*m in pictures of 100x and 20 *μ*m in pictures of 400x.

To further investigate the influence of CVP and GF on AD mice in the histological level, hippocampus in the brain tissue was applied for H&E staining. As shown in Figure 3, cells in the normal group kept structure integrity and were arrayed in order. In the model group, the volume of the cells decreased and the stain of the cells was dark. In addition, shrinkage and necrosis were observed in scattered cells. In the CVP group, two in three were found with hyperchromatic nuclei in the hippocampus and nuclear shrinkage. In the GF group, all samples showed part of hyperchromatic nuclei in the hippocampus, but the hyperchromatic degree is relatively lower than that in the model group. In the combination treatment group, only one sample was observed with hyperchromatic nuclei and with relative lower degree. Taken together, according to H&E staining, the combination treatment of CVP and GF significantly alleviate the pathological degree in the brain tissue of AD mice.

### 3.3. CVP and GF Alleviate Inflammation and Enhance Antioxidation Activity in the CNS of AD Mice

**Figure 4:**
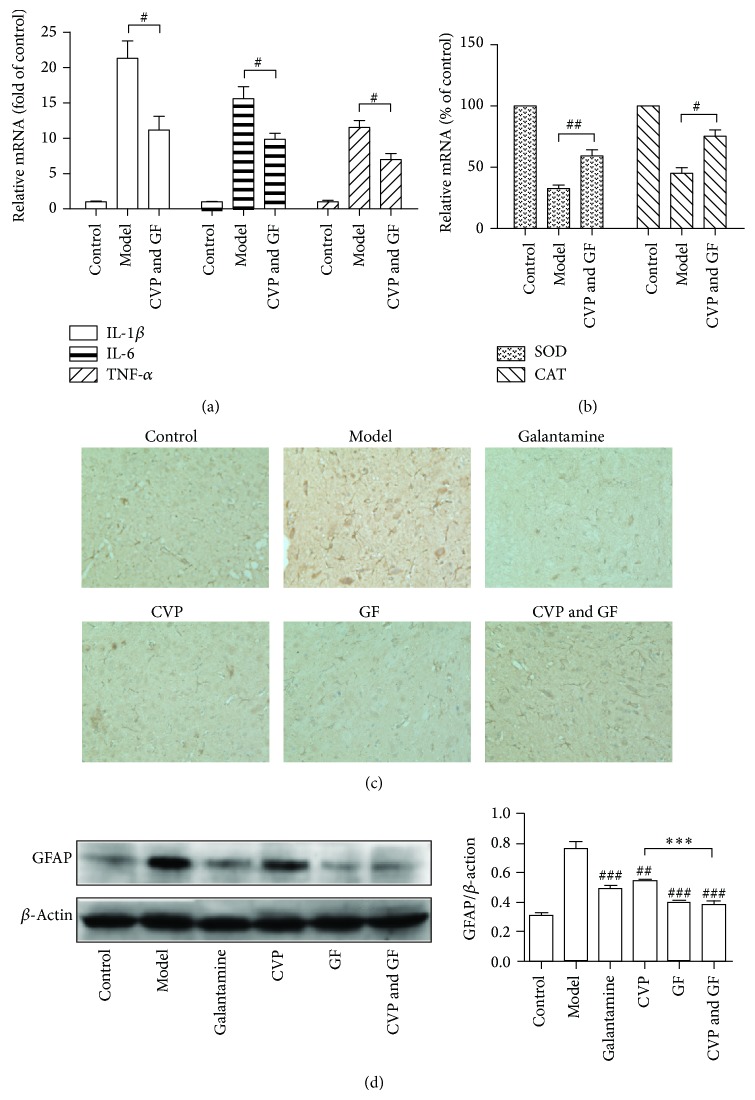
Examination of the inflammation level and antioxidative capacity in brain tissue. (a) The mRNA levels of IL-1*β*, IL-6, and TNF-*α* in brain tissue were analyzed by Q-PCR. (b) The mRNA levels of SOD and CAT in the brain tissue were analyzed by Q-PCR. Data are mean ± SEM of three independent experiments. ^#^ *P* < 0.05; ^##^ *P* < 0.01. (c) Paraffin sections of hippocampus from brain tissues of AD model mice were analyzed by immunohistochemistry for GFAP. GFAP is indicated in yellow. Data are representative of each group (*n* = 8). (d) The expression of GFAP protein in the brain was determined by Western blotting. The relative band density of GFAP was analyzed using Image J. Data are mean ± SEM of three independent experiments. ^***^ *P* < 0.005; ^##^ *P* < 0.01; ^###^ *P* < 0.005* versus* model group.

To evaluate the level of inflammation in the brain tissue of AD mice, the expression levels of interleukin-1 beta (IL-1*β*), IL-6, and tumor necrosis factor (TNF-*α*), the three most common inflammatory factors produced by microglia, were determined (Figure 4(a)). The results showed that the mRNA levels of IL-1*β*, IL-6, and TNF-*α* were decreased significantly by CVP and GF, indicating that inflammation in AD mice was inhibited by treatment with these compounds. The expression of superoxide dismutase (SOD) and catalase (CAT) was also investigated to evaluate the antioxidative activity in the CNS of AD mice (Figure 4(b)). SOD and CAT are important for the elimination of ROS. The resultant data suggested that the combinational use of CVP and GF improved antioxidative activity in the brain tissue of AD mice.

In addition, GFAP was detected by both immunohistochemistry and Western blotting. GFAP is a specific biomarker of activated astrocytes, and the activation of astrocytes is usually accompanied with inflammation [31]. The IHC results showed that there were an increased number of GFAP-positive cells in the model group and that these cells were stained more strongly than in the normal group. Compared with the model group, the numbers of GFAP-positive cells in each drug treatment group were decreased by different amounts (Figure 4(c)). To further confirm the results, GFAP expression was also determined by Western blotting (Figure 4(d)). Grayscale analysis showed that the expression of GFAP was decreased to the greatest extent in the GF plus CVP combination treatment group, indicating that CVP and GF inhibit the activation of astrocytes. Taken together, it can be concluded that CVP and GF exhibit potential anti-inflammatory and antioxidative effects in the brain tissue of AD mice.

## 4. Discussion and Conclusion

AchE inhibitors are the first anti-AD drugs to be approved by the FDA, and they are also the first and the most useful drugs to have been used in the clinical treatment of AD [32]. Previous studies have indicated that AD is a complex disease that is related to senile plaques and neurofibrillary tangles [33]. It appears that single-target drugs are only suitable for mild or moderate AD patients. Therefore, an increasing level of interest has been taken in the field of natural medicines. Here, we discussed the anti-AD effect of the combination of CVP and GF.

CVP and GF are two natural extracts that have been widely used in traditional medicine. In our study, it was observed that treatment with CVP or GF individually resulted in only slight improvements in the following metrics: spatial memory ability in an MWM test, the number of correct responses in a Y-maze test, the probability of errors made in step-through test, and cell abnormalities in the brain tissue. Further study of the CVP and GF combination showed that the formula exhibited a better curative effect on AD mice. The significant differences of *P* value* versus* model group in CVP & GF group were all smaller than in single-drug treatment group. In addition, the therapeutic effects of CVP & GF were better than CVP or GF in the probe trial and step-through test. Above data indicated that CVP and GF have synergistic effects with respect to improving the learning and memory abilities of AD mice.

Although there are several hypotheses regarding the cause of most cases of AD that have been proposed, such as genetic predispositions, reduced acetylcholine synthesis, amyloid- and Tau-related accumulation, this matter is still the subject of debate [34]. The damage that occurs in neurons is a long-term process that eventually leads to the development of AD. Multiple studies suggest that inflammation plays an important role in AD [35]. Amyloid-*β* deposits activate microglia to produce a variety of inflammatory factors, including, IL-1*β*, IL-6, and TNF-*α*, resulting in neurotoxicity [4].

Firstly, we aim to illustrate that the anti-inflammatory defenses of neurons can be targeted in future strategies for the treatment of AD. The mRNA levels of IL-1*β*, IL-6, and TNF-*α* in the brain tissue of AD mice were detected. Our results showed that the expression of all the three inflammatory factors was inhibited by CVP and GF. As previously described, GFAP is a marker of activated astrocytes which usually contributes to the development of amyloid plaques. The activation of astrocyte is also accompanied with inflammation [4, 5]. Here, we found that the expression of GFAP in brain tissue was significantly downregulated by the treatment of CVP and GF. Taken together, our data indicated that the activation of astrocytes and inflammation in the brain tissue of AD mice were effectively inhibited by the combined treatment.

In addition to inflammation, cellular oxidation is also primary cause of aging. Cellular oxidation is characterized by the accumulation of reactive oxygen species, such as O_2_^−^ and H_2_O_2_. Antioxidant enzymes, such as SOD and CAT, catalyze the decomposition of ROS. The decreased mRNA levels of SOD and CAT in AD mice were elevated by combination treatment of CVP and GF, suggesting increased antioxidative activity in the brain tissues of AD mice.

**Figure 5:**
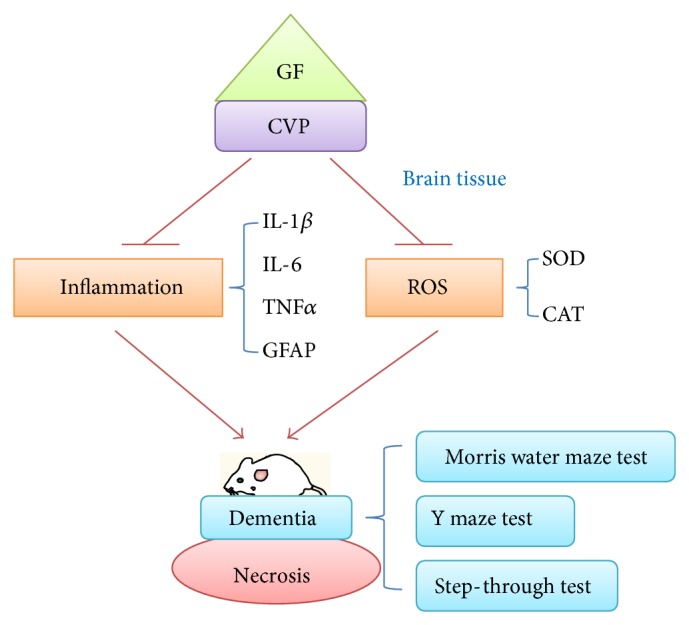
A scheme for the synergistic effects of CVP and GF on dementia.

In conclusion, the combination of CVP and GF shows a synergistic effect with respect to improving the memory of D-gals and AlCl_3_-induced AD mouse model (Figure 5). The elevated inflammation and oxidation levels in the brain tissue of AD mice were found to be alleviated by the combination treatment. Our research provides a new insight into the treatment of dementia.

## Supplementary Material

Statistical analysis was performed to determine the exact probability levels versus the model group unless otherwise specified in the corresponding figures
