# Supplementary material for: The Synergistic Beneficial Effects of Ginkgo Flavonoid and Coriolus versicolor Polysaccharide for Memory Improvements in a Mouse Model of Dementia
Source: Evid Based Complement Alternat Med. 2015 Mar 2;2015:128394. doi: 10.1155/2015/128394 (PMC4364004; doi:10.1155/2015/128394)
Supplement: Supplementary file 1 — Statistical analysis was performed to determine the exact probability levels versus the model group unless otherwise specified in the corresponding figures [file 128394.f1.pdf]

Supplementary table. 95% confidence interval

group	figure 1A			figure 1B	
	D1	D2	D3	percent time	passing times
Control	-	-	-	-	-
Model vs Control	-29.93 to -2.156	-44.37 to -16.39	-40.21 to -18.57	18.24 to 31.63	1.778 to 3.722
Galanthamine	6.221 to 36.43	5.250 to 36.36	8.166 to 34.20	-25.92 to -10.88	-3.360 to -1.640
CVP	5.782 to 32.34	3.604 to 34.07	6.279 to 33.34	-26.47 to -13.34	-1.535 to 0.03499
GF	5.605 to 31.89	4.297 to 35.39	9.147 to 34.41	-26.08 to -11.65	-2.949 to -0.5509
CVP & GF	7.916 to 33.09	6.772 to 38.86	9.303 to 34.63	-26.97 to -14.44	-3.450 to -0.7998
CVP & GF vs CVP	-11.58 to 14.47	-10.71 to 18.67	-12.23 to 16.55	-7.372 to 5.770	-2.716 to -0.03438
CVP & GF vs GF	-11.13 to 14.64	-12.04 to 17.99	-13.36 to 13.74	-9.060 to 5.375	-1.993 to 1.243

95% confidence interval

group	figure 2A	figure 2B	
	the right entries	latency	error numbers
Control	-	-	-
Model vs Control	2.407 to 4.993	29.49 to145.0	-4.154 to -2.096
Galanthamine	-4.495 to -1.105	-104.0 to 4.454	2.017 to 3.983
CVP	-3.344 to -0.2561	-76.84 to 3.585	1.187 to 3.313
GF	-3.968 to -0.03158	-84.27 to -8.228	1.476 to 3.524
CVP & GF	-4.125 to -1.075	-129.4 to -38.63	1.327 to 3.673
CVP & GF vs CVP	-2.318 to 0.7183	-86.34 to -8.408	-0.7735 to 1.274
CVP & GF vs GF	-2.548 to 1.348	-74.45 to -1.045	-0.9825 to 0.9825
